# Testing *DAT1* and *DRD4* Genes in Attention Deficit Hyperactivity Disorder Using a Wide Spectrum of Neurocognitive Batteries

**DOI:** 10.1002/jdn.70133

**Published:** 2026-04-27

**Authors:** Gül Ünsel‐Bolat, Hilmi Bolat, Sema Yıldırım, Semiha Özgül, Akın Tahıllıoğlu, Kemal Utku Yazıcı, Ali Bacanlı, Erhan Parıltay, Duygu Aygüneş‐Jafari, Buket Kosova, Luis Augusto Rohde, Haluk Akın, Eyüp S. Ercan

**Affiliations:** ^1^ Department of Child and Adolescent Psychiatry Balıkesir University Balıkesir Turkey; ^2^ Department of Medical Genetics Balıkesir University Balıkesir Turkey; ^3^ Biostatistics and Medical Informatics Ege University İzmir Turkey; ^4^ Department of Child and Adolescent Psychiatry Bakırçay University İzmir Turkey; ^5^ Department of Child and Adolescent Psychiatry Fırat University Elazığ Turkey; ^6^ Department of Child and Adolescent Psychiatry Private Practice İzmir Turkey; ^7^ Department of Medical Genetics Ege University İzmir Turkey; ^8^ Medical Biology Ege University İzmir Turkey; ^9^ Department of Psychiatry Universidade Federal do Rio Grande do Sul Porto Alegre Brazil; ^10^ ADHD and Developmental Psychiatry Programs Hospital de Clinicas de Porto Alegre Porto Alegre Brazil; ^11^ National Institute of Developmental Psychiatry for Children and Adolescents Porto Alegre Brazil; ^12^ Department of Child and Adolescent Psychiatry Ege University İzmir Turkey

**Keywords:** ADHD, *DAT1*, dopamine, *DRD4*, genotype, neurocognitive function, VNTR

## Abstract

Attention‐deficit/hyperactivity disorder (ADHD) is a neurodevelopmental condition characterized by marked heterogeneity in cognitive functioning. This study aimed to examine the associations between polymorphisms in the *DAT1* and *DRD4* genes and neurocognitive performance in children and adolescents with ADHD. A total of 336 participants aged 6–18 years (244 with ADHD and 92 healthy controls) were included. Variable number tandem repeat (VNTR) polymorphisms in the 3′ UTR of *DAT1* and exon 3 of *DRD4* were genotyped. Neurocognitive performance was assessed using standardized scores derived from the CNS Vital Signs battery. Associations between genotypes and cognitive domains were examined using analysis of covariance (ANCOVA), adjusting for age and gender. Homozygosity for the *DRD4* 4‐repeat allele was significantly associated with poorer cognitive flexibility, whereas a trend‐level difference was observed for complex attention. In contrast, *DAT1* 10R/10R homozygosity and *DRD4* 7‐repeat allele carriage were not associated with significant differences in reaction time, complex attention or cognitive flexibility. These findings suggest that *DRD4*, rather than *DAT1*, may represent a more salient dopaminergic genetic marker of executive dysfunction in ADHD. The results underscore the domain‐specific and modest nature of genetic influences on cognition and highlight the importance of integrating genetic markers with cognitive endophenotypes to better characterize heterogeneity in ADHD.

## Graphical abstract

This study examined *DAT1* and *DRD4* VNTR polymorphisms and neurocognitive performance in 336 children and adolescents with ADHD and controls using the CNS Vital Signs battery. *DAT1* 10R/10R showed no significant effects after adjustment, whereas *DRD4* 4R/4R was associated with reduced cognitive flexibility.

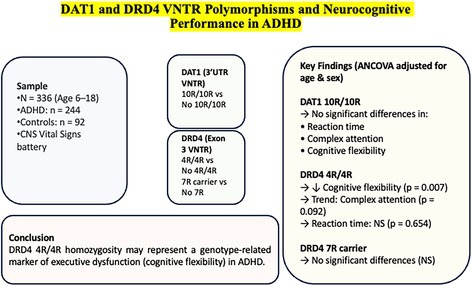

## 1 Introduction

Attention‐deficit/hyperactivity disorder (ADHD) is a neurodevelopmental condition characterized by developmentally inappropriate levels of distractibility, excessive motor activity and impulsive behaviour. It represents one of the most frequently encountered psychiatric conditions during school‐age years (Thomas et al. 2015). Recent epidemiological data indicate that the global prevalence among children of school age ranges between approximately 5% and 7% (Thomas et al. 2015; Polanczyk et al. 2007).

Substantial evidence from twin and family studies indicates that ADHD has a strong genetic basis, with heritability estimates ranging from 60% to 90% (Faraone and Larsson 2019). Among the neurobiological systems implicated, the dopaminergic system has drawn significant interest, particularly due to its regulatory role in attentional control, motivation and executive functions (Faraone et al. 2024).

Within this system, two of the most thoroughly examined candidate genes are the dopamine transporter gene (*DAT1*, also referred to as SLC6A3) and the dopamine D4 receptor gene (*DRD4*) (Cortese 2012; Faraone et al. 2014). Both genes are believed to modulate synaptic dopamine availability, thereby influencing cognitive domains relevant to ADHD symptomatology (Faraone et al. 2024).

While many studies have explored the relationship between these genes and the presence of ADHD (Akutagava‐Martins et al. 2016), their specific influence on neurocognitive characteristics—often referred to as cognitive endophenotypes—is not fully elucidated. Given the clinical heterogeneity observed among individuals with ADHD, evaluating how these genetic variants relate to performance in targeted cognitive areas may clarify underlying biological pathways and help reduce phenotypic variability.

This study sought to investigate the association between specific polymorphisms in the *DAT1* and *DRD4* genes and cognitive performance among children and adolescents with ADHD. By examining defined VNTR variants and their link to neurocognitive subdomains, we aim to contribute to a more precise understanding of the disorder's neurobiological structure.

## 2 Methods

This study was conducted using a cross‐sectional design and included participants recruited from a university‐based outpatient clinic for child and adolescent psychiatry. ADHD diagnoses were established according to DSM‐5 criteria, while symptom severity assessment was supported using the Turkish version of the Turgay ADHD Rating Scale based on DSM‐IV symptom definitions.

A total of 336 participants were included: 244 individuals diagnosed with ADHD and 92 age‐matched healthy controls. All ADHD participants were assessed at their initial diagnostic evaluation and were treatment‐naive, with no history of stimulant or other psychotropic medication use prior to neurocognitive testing. Cognitive performance was evaluated using the Central Nervous System–Vital Signs (CNS Vital Signs; CNS–VS) test battery, which was administered to all subjects. Standardized scores derived from CNS–VS domains were used in the analyses.

### 2.1 Inclusion Criteria

Eligible participants were aged between 6 and 18 years, lived with their families, attended formal education and did not present with intellectual disability, neurological disorders, history of significant head trauma, perinatal complications or substance use issues. Participants also needed to demonstrate clear ADHD symptomatology in line with diagnostic criteria.

Prior to the study, ethical approval was obtained from Ege University (Protocol No: 16‐5.2/2, Date: 27 July 2016). Written informed consent was obtained from all caregivers of participating children.

### 2.2 Measures Employed in the Study

#### 2.2.1 Computerized Neurocognitive Assessment—CNS–VS

The Computerized Neurocognitive Assessment–Vital Signs (CNS–VS) is a digital test battery that evaluates various cognitive domains, including executive functions. It comprises several tasks administered in a fixed order without breaks. From these tasks, six primary composite scores are derived to summarize neurocognitive functioning (Gualtieri and Johnson 2006).

##### 2.2.1.1 Main Composite Scores

- **Total memory**: Reflects both verbal and visual memory abilities, derived from memory task performances.
- **Reaction time**: Assesses response latency during tasks involving both simple and complex stimuli.
- **Complex attention**: Captures the capacity to focus, maintain attention and inhibit automatic responses under demanding conditions.
- **Cognitive flexibility**: Measures the ability to adapt cognitive strategies in response to changing task rules or stimuli.
- **Psychomotor speed**: Evaluates fine motor performance and visual‐motor integration based on speed‐oriented tasks.
- **Neurocognitive index**: A summary score computed from the above five domains, offering a general indication of cognitive status (Gualtieri and Johnson 2006).

##### 2.2.1.2 Brief Description of Each Subtest

- **Verbal and Visual Memory Tests (VBM, VIM)**: Involve recognition and recall of word lists and geometric figures, respectively, to assess immediate and delayed memory performance.
- **Finger Tapping Test (FTT)**: Measures motor speed by recording the number of taps produced with each hand in a fixed time.
- **Symbol Digit Coding Test (SDC)**: Requires matching symbols to digits using a provided key, emphasizing processing speed and attention.
- **Stroop Test (ST)**: Involves naming the colour of words or symbols that may conflict with their semantic meaning, targeting inhibitory control and reaction speed.
- **Shifting Attention Test (SAT)**: Evaluates cognitive flexibility by requiring the participant to alternate between different matching rules (e.g., shape vs. colour) under time pressure.
- **Continuous Performance Test (CPT)**: Assesses sustained attention by measuring the accuracy of responses to target and nontarget stimuli over a prolonged period (Gualtieri and Johnson 2006).

### 2.3 Genotyping

Variable number tandem repeat (VNTR) polymorphisms within the *DAT1* and *DRD4* genes were the primary focus of genotyping. Specifically, the 40‐base pair VNTR in the 3′ UTR of *DAT1* and the 48‐base pair VNTR in exon 3 of *DRD4* were analysed (Faraone and Larsson 2019; Ebstein 2006).

Genomic DNA was extracted from either saliva or peripheral blood using commercially available kits, adhering to the manufacturers' protocols. PCR amplification targeted the respective VNTR loci using primer sequences whose validity had been confirmed in earlier studies (Bolat et al. 2019). Amplified products were resolved via agarose gel electrophoresis and visualized under UV illumination following ethidium bromide staining. All genotyping procedures were carried out under blinded conditions.

To ensure genotyping accuracy, positive and negative controls were included in each PCR run, and a random subset of samples was re‐genotyped for quality control purposes, yielding 100% concordance.

### 2.4 Data Analysis

Descriptive statistical methods were employed to summarize sample characteristics. Categorical variables were presented as counts and percentages, while continuous variables were summarized using means and standard deviations.

Associations between *DAT1* and *DRD4* genotypes and neurocognitive performance were examined using analysis of covariance (ANCOVA), adjusting for age and gender as covariates. For the *DAT1* gene, participants homozygous for the 10‐repeat allele (10R/10R) were compared with all other genotypes. For the *DRD4* gene, two genetic models were tested: (i) homozygosity for the 4‐repeat allele (4R/4R) versus non‐homozygous individuals and (ii) carriage of the 7‐repeat allele versus noncarriers.

Neurocognitive outcomes included standardized scores for reaction time, complex attention and cognitive flexibility derived from the CNS Vital Signs battery. Effect sizes were estimated using partial eta‐squared (η^2^). A two‐tailed *p*‐value of less than 0.05 was considered statistically significant.

Although multiple cognitive domains were examined, the primary analyses were hypothesis‐driven and focused on executive function–related domains (reaction time, complex attention and cognitive flexibility), thereby reducing the risk of inflated Type I error.

All statistical analyses were conducted using IBM SPSS Statistics (version 31.0.1.0 (49)).

## 3 Results

The study included 336 children aged between 6 and 18 years (mean age: 10.65 ± 2.01), of whom 68.2% were male (*n* = 229). While 27.4% (*n* = 92) of the participants were classified as healthy controls without any psychiatric diagnosis, the remaining subjects were diagnosed with ADHD (*n* = 244).

Neurocognitive test performances were examined in relation to *DAT1* and *DRD4* genotypes using CNS Vital Signs–derived standardized scores. All analyses were conducted using ANCOVA, adjusting for age and gender.

### 3.1 Neurocognitive Test Results of DAT1 Gene Variants

Participants homozygous for the 10‐repeat allele of the *DAT1* gene (10R/10R) were compared with all other genotypes. No statistically significant differences were observed between groups across reaction time, complex attention or cognitive flexibility domains after controlling for age and gender.

Specifically, no significant group differences were found for reaction time (*p* = 0.720, partial η^2^ = 0.001), complex attention (*p* = 0.499, partial η^2^ = 0.002) or cognitive flexibility (*p* = 0.966, partial η^2^ = 0.000). These findings indicate that *DAT1* 10R/10R homozygosity was not associated with measurable impairments in neurocognitive performance in the present sample.

Descriptive statistics and ANCOVA results for *DAT1* 10R/10R homozygosity are presented in Table 1.

**TABLE 1:** *DAT1* 10R/10R homozygosity and neurocognitive outcomes (ANCOVA adjusted for age and gender).

Outcome (standard score)	No 10R/10R mean ± SD (*n*)	10R/10R mean ± SD (*n*)	*p*	Partial η^2^
Reaction time	72.1161 ± 24.99324 (*n* = 112)	73.8372 ± 23.72656 (*n* = 129)	0.720	0.001
Complex attention	76.8929 ± 23.40068 (*n* = 112)	75.9380 ± 25.07543 (*n* = 129)	0.499	0.002
Cognitive flexibility	83.2342 ± 14.74015 (*n* = 111)	83.5234 ± 14.82016 (*n* = 128)	0.966	0.000

*Note:* Participants homozygous for the 10‐repeat allele of the *DAT1* gene (10R/10R) were compared with all other genotypes. Neurocognitive performance was assessed using standardized scores derived from the CNS Vital Signs battery. Group differences were tested using ANCOVA, adjusting for age and gender as covariates. Values are presented as mean ± standard deviation. *p* values and partial eta‐squared (η^2^) were derived from the tests of between‐subjects effects table.

### 3.2 Neurocognitive Test Results of DRD4 Gene Variants

Participants homozygous for the 4‐repeat allele of the *DRD4* gene (4R/4R) exhibited significantly poorer performance in cognitive flexibility compared with those without 4R/4R homozygosity (*p* = 0.007, partial η^2^ = 0.030), after adjusting for age and gender.

In addition, a trend‐level difference was observed in complex attention scores, with 4R/4R carriers showing lower performance than noncarriers (*p* = 0.092, partial η^2^ = 0.012).

No statistically significant group differences were detected in reaction time (*p* = 0.654, partial η^2^ = 0.001). Descriptive statistics and ANCOVA results for *DRD4* 4R/4R homozygosity are presented in Table 2.

**TABLE 2:** *DRD4* 4R/4R homozygosity and neurocognitive outcomes (ANCOVA adjusted for age and gender).

Outcome (standard score)	No 4R/4R mean ± SD (*n*)	4R/4R mean ± SD (*n*)	*p*	Partial η^2^
Cognitive flexibility	86.6170 ± 14.00315 (*n* = 94)	81.2966 ± 14.89506 (*n* = 145)	0.007	0.030
Complex attention	79.8298 ± 19.09548 (*n* = 94)	74.1769 ± 26.89113 (*n* = 147)	0.092	0.012
Reaction time	72.2021 ± 25.97417 (*n* = 94)	73.5714 ± 23.22050 (*n* = 147)	0.654	0.001

*Note:* Participants homozygous for the 4‐repeat allele of the *DRD4* gene (4R/4R) were compared with non‐homozygous individuals. Neurocognitive performance was assessed using standardized scores derived from the CNS Vital Signs battery. Group differences were tested using ANCOVA, adjusting for age and gender as covariates. Values are presented as mean ± standard deviation. *p* values and partial eta‐squared (η^2^) were derived from the tests of between‐subjects effects table.

Participants carrying the 7‐repeat allele of the *DRD4* gene were compared with noncarriers. No statistically significant differences were found between groups in cognitive flexibility (*p* = 0.213, partial η^2^ = 0.007), complex attention (*p* = 0.090, partial η^2^ = 0.012) or reaction time (*p* = 0.111, partial η^2^ = 0.011) after controlling for age and gender.

These findings indicate that *DRD4* 7‐repeat allele carriage was not associated with significant alterations in neurocognitive performance in the domains assessed.

Descriptive statistics and ANCOVA results for *DRD4* 7‐repeat allele carriage are presented in Table 3.

**TABLE 3:** *DRD4* 7‐repeat allele carriage and neurocognitive outcomes (ANCOVA adjusted for age and gender).

Outcome (standard score)	No 7R mean ± SD (*n*)	7R carrier mean ± SD (*n*)	*p*	Partial η^2^
Cognitive flexibility	82.8259 ± 15.22907 (*n* = 201)	86.3684 ± 11.63968 (*n* = 38)	0.213	0.007
Complex attention	75.1084 ± 25.49720 (*n* = 203)	83.1842 ± 14.62293 (*n* = 38)	0.090	0.012
Reaction time	74.0394 ± 23.75699 (*n* = 203)	67.6842 ± 26.64695 (*n* = 38)	0.111	0.011

*Note:* Participants carrying the 7‐repeat allele of the *DRD4* gene were compared with noncarriers. Neurocognitive performance was assessed using standardized scores derived from the CNS Vital Signs battery. Group differences were tested using ANCOVA, adjusting for age and gender as covariates. Values are presented as mean ± standard deviation. *p* values and partial eta‐squared (η^2^) were derived from the tests of between‐subjects effects table.

## 4 Discussion

### 4.1 Association of DAT1 Gene Variants With Neurocognitive Test Results

Contrary to our initial hypothesis, *DAT1* 10R/10R homozygosity was not associated with significant differences in reaction time, complex attention or cognitive flexibility after controlling for age and gender. These null findings suggest that, within the present sample, *DAT1* 10‐repeat homozygosity may not exert a robust or domain‐specific influence on neurocognitive performance as measured by CNS Vital Signs.

Previous studies have reported associations between the *DAT1* 10R/10R genotype and altered dopamine transporter expression, potentially leading to reduced synaptic dopamine availability in striatal regions (Klein et al. 2017; Cheon et al. 2005). Moreover, pharmacogenetic investigations have suggested attenuated stimulant response among individuals carrying this genotype (Froehlich et al. 2011), and neuroimaging research has demonstrated functional alterations in working memory networks (Pineau et al. 2019). The absence of significant effects in the current study may reflect the small effect sizes of *DAT1* variants on cognition, phenotypic heterogeneity within ADHD or limited sensitivity of the CNS Vital Signs battery to subtle dopaminergic modulation.

Our null findings for *DAT1* 10R/10R are consistent with recent evidence indicating that the phenotypic expression of dopaminergic variants may be domain‐specific (Faraone et al. 2024). While *DAT1* is primarily associated with subcortical dopaminergic tonicity and striatal transporter density (Klein et al. 2017), executive functions like cognitive flexibility may be more sensitive to *DRD4*‐mediated modulation in the prefrontal cortex (Faraone et al. 2024).

Taken together, these findings indicate that *DAT1* variability alone may be insufficient to account for individual differences in higher‐order neurocognitive performance, emphasizing the importance of considering polygenic models and gene–environment interactions in future studies.

### 4.2 Association of DRD4 Gene Variants With Neurocognitive Test Results

In contrast to *DAT1*, homozygosity for the *DRD4* 4‐repeat allele (4R/4R) was significantly associated with poorer cognitive flexibility, even after adjustment for age and gender. A trend‐level difference was also observed for complex attention, whereas no significant group differences emerged for reaction time. These findings suggest that the *DRD4* 4R/4R genotype may selectively influence executive functions related to set‐shifting and attentional control rather than global processing speed.

The *DRD4* receptor plays a critical role in prefrontal dopaminergic signaling and modulation of executive control circuits (Bellgrove et al. 2008). Prior studies have linked the 4R genotype to variations in methylphenidate treatment response and to altered performance on attention tasks, such as the Continuous Performance Test (Gizer et al. 2009; Opmeer et al. 2010). The present findings extend this literature by demonstrating a specific association between *DRD4* 4R/4R homozygosity and reduced cognitive flexibility, a core component of executive functioning that underlies adaptive behavioural regulation and problem‐solving.

Participants carrying the *DRD4* 7‐repeat allele did not exhibit statistically significant differences in cognitive flexibility, complex attention or reaction time compared with noncarriers. Although a trend toward higher complex attention scores was observed among 7R carriers, this effect did not reach conventional levels of statistical significance. These results are broadly consistent with prior reports suggesting modest and context‐dependent effects of the 7R allele on cognitive phenotypes (Ebstein 2006), and they underscore the heterogeneity of *DRD4*‐related cognitive associations.

Collectively, these findings indicate that *DRD4* genetic variability may moderate neurocognitive performance in ADHD in a domain‐specific manner, with 4R/4R homozygosity emerging as a more salient marker of executive dysfunction than 7R allele carriage.

### 4.3 Limitations

Several limitations of this study should be acknowledged. First, although the overall sample size was moderate, subgroup sizes for specific genotypes were relatively small, which may have limited statistical power to detect subtle genotype–cognition associations.

Second, although medication status at the time of neurocognitive testing was not recorded as a separate variable in the dataset, all ADHD participants were assessed at their initial diagnostic evaluation and were treatment‐naive, with no prior exposure to stimulant or other psychotropic medication. Therefore, acute pharmacological effects are unlikely to have confounded the observed genotype–cognition associations. Nevertheless, future studies should prospectively document medication history in a standardized manner, as medication exposure may become an important confounder in broader clinical samples.

Third, although ADHD diagnoses were established according to DSM‐5 criteria, symptom assessment and clinical follow‐up were supported using the validated Turkish version of the Turgay ADHD Rating Scale, which is anchored to DSM‐IV symptom definitions. While core ADHD symptom domains remain largely comparable across diagnostic systems, minor differences in diagnostic thresholds and subtype conceptualizations may limit cross‐study comparability. Nevertheless, the use of this widely applied instrument ensured standardized symptom quantification and enhanced ecological validity within the local clinical context.

Fourth, multiple neurocognitive domains were examined across several genetic models. Although the primary analyses were hypothesis‐driven and focused on executive function–related domains, the possibility of Type I error due to multiple comparisons cannot be entirely excluded. Nevertheless, the consistency of effect direction and the domain‐specific pattern observed for *DRD4* 4R/4R homozygosity support the robustness of the main finding.

Fifth, neurocognitive performance was assessed exclusively using the CNS Vital Signs battery. While this instrument captures a broad range of cognitive domains, it may not fully reflect real‐world executive functioning or ecologically valid behavioural outcomes.

Finally, the cross‐sectional design precludes causal inference, and longitudinal patterns of gene–cognition associations remain unexplored.

### 4.4 Clinical and Research Implications

The present findings underscore the importance of integrating genetic markers with cognitive endophenotypes to better characterize heterogeneity in ADHD. In particular, *DRD4* 4R/4R homozygosity was associated with impaired cognitive flexibility, a domain central to adaptive executive control. In contrast, *DAT1* 10R/10R homozygosity was not associated with measurable neurocognitive impairments, highlighting the domain‐ and gene‐specific nature of dopaminergic genetic effects.

Previous meta‐analyses have consistently shown that individuals with ADHD exhibit mild to moderate deficits in problem‐solving, sustained attention and working memory (Schoechlin and Engel 2005). Verbal working memory, in particular, has been identified as a core cognitive vulnerability in children and adolescents with ADHD (Ramos et al. 2020). The current results suggest that underlying genetic variation in *DRD4* may contribute to executive dysfunction, whereas *DAT1* effects on cognition may be weaker or more context‐dependent.

From a clinical perspective, characterizing neurocognitive profiles based on genotype may support the development of individualized treatment strategies. Identifying patients with genotypes linked to executive dysfunction may inform more targeted cognitive or pharmacological interventions. Such genotype‐informed approaches may be particularly valuable in pharmacogenetics and in optimizing non‐pharmacological treatment planning.

It is important to acknowledge that ADHD is fundamentally polygenic. Recent large‐scale genomic studies (Demontis et al. 2023; Faraone et al. 2024) suggest that individual candidate genes like *DRD4* explain only a small fraction of the total heritability. Future research should increasingly focus on polygenic risk architectures to better capture the neurocognitive heterogeneity of the disorder.

Future research should focus on validating these associations in larger and more diverse cohorts, incorporating comprehensive medication data and exploring how genetic and cognitive profiles jointly predict long‐term functional outcomes. Such efforts may ultimately facilitate the development of more personalized and effective care models for individuals with ADHD.

## 5 Conclusion

The present study provides evidence that genetic variability in the dopaminergic system may contribute to domain‐specific differences in neurocognitive functioning in children and adolescents with ADHD. In particular, homozygosity for the *DRD4* 4‐repeat allele was associated with impaired cognitive flexibility, highlighting a potential role for *DRD4* in executive control processes.

In contrast, *DAT1* 10R/10R homozygosity and *DRD4* 7‐repeat allele carriage were not associated with significant alterations in reaction time, complex attention or cognitive flexibility. These findings underscore the heterogeneity and modest effect sizes of dopaminergic genetic influences on cognition in ADHD.

Overall, the results suggest that *DRD4*, rather than *DAT1*, may represent a more salient genetic marker of executive dysfunction in ADHD. Future studies incorporating larger samples, longitudinal designs and comprehensive medication data are warranted to further clarify the role of dopaminergic genes in shaping neurocognitive phenotypes and to support the development of genotype‐informed approaches to assessment and intervention.

## Author Contributions

Gül Ünsel‐Bolat, Sema Yıldırım, Akın Tahıllıoğlu, Kemal Utku Yazıcı, Ali Bacanlı, Luis A Rohde and Eyüp S Ercan provided psychiatric evaluation. Hilmi Bolat provided genetic evaluation. Gul Unsel‐Bolat and Hilmi Bolat designed the study. Hilmi Bolat, Haluk Akın, Erhan Parıltay, Duygu Aygüneş‐Jafari and Buket Kosova made genetic analyses. Semiha Ozgul provided statistical analyses. Gul Unsel‐Bolat, Hilmi Bolat, Semiha Ozgul and Sema Yıldırım wrote the manuscript.

## Funding

This study was supported by the Ege University Scientific Research Projects Coordinator (TTU‐2018‐20009).

## Ethics Statement

This study was conducted in accordance with the ethical principles of the Declaration of Helsinki. Ethics approval was granted by the Ege University Clinical Research Ethics Committee in Türkiye (Approval No: 16‐5.2/2, Date: 27 July 2016). Written informed consent was obtained from all participants' parents or legal guardians prior to data collection.

## Conflicts of Interest

The authors declare no conflicts of interest.

## Data Availability

Data underlying the findings of this study are not publicly available due to ethical and privacy considerations. However, anonymized data can be made available from the corresponding author upon reasonable request.
